# Supplementary material for: Nicotine in Combination with SARS-CoV-2 Affects Cells Viability, Inflammatory Response and Ultrastructural Integrity
Source: Int J Mol Sci. 2022 Aug 22;23(16):9488. doi: 10.3390/ijms23169488 (PMC9409480; doi:10.3390/ijms23169488)

Supplemental figures

Supplementary **Figure S1** - Autophagosomes with intact content as supplement to figure 4

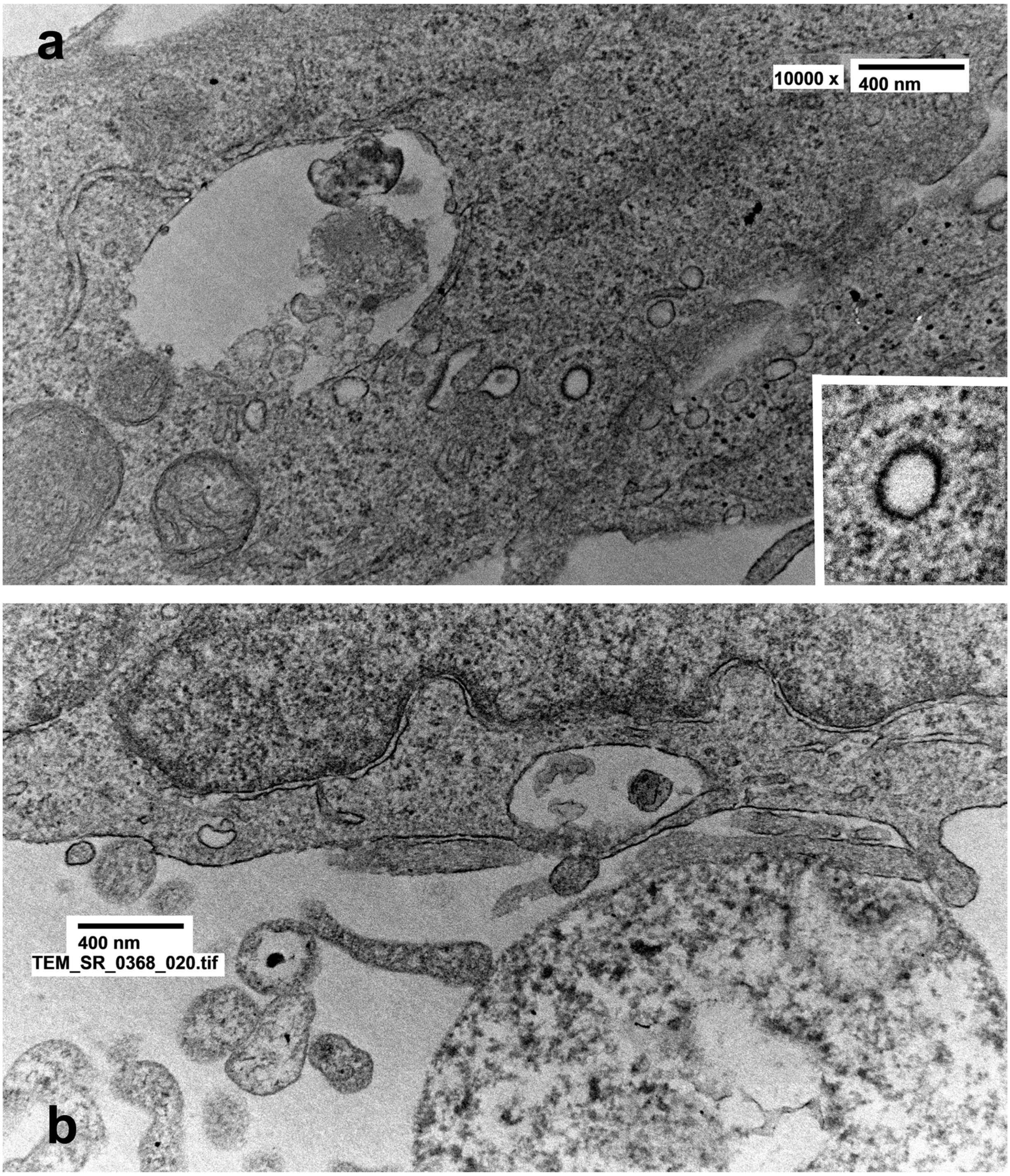

Supplementary **Figure S2** - Evidences of membrane pores (c,d) as typically seen in pyroptosis. Compare with intact control membrane (a,b).

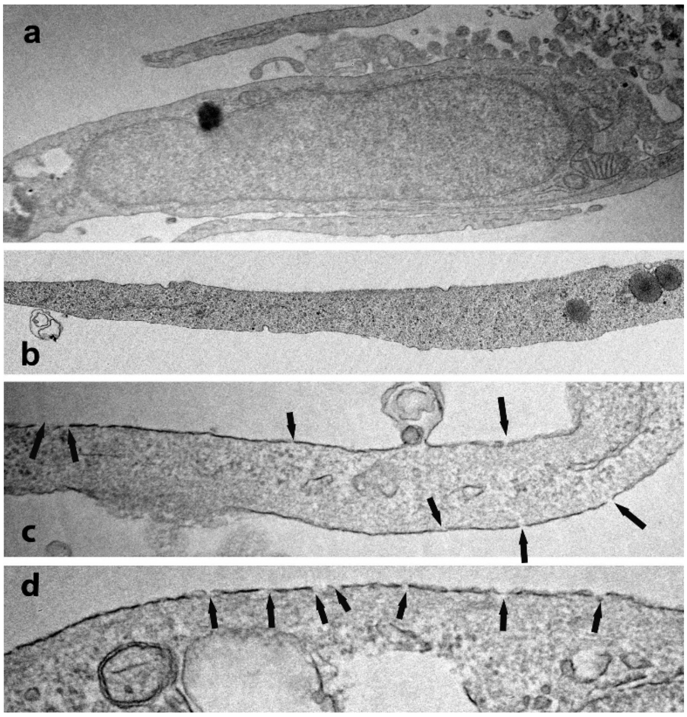

Supplement: Supplementary file 1 [file ijms-23-09488-s001.zip › ijms-1860118-supplementary.pdf]
